# Advances in keratinocyte delivery in burn wound care

**DOI:** 10.1016/j.addr.2017.06.012

**Published:** 2018-01-01

**Authors:** Britt ter Horst, Gurpreet Chouhan, Naiem S. Moiemen, Liam M. Grover

**Affiliations:** a School of Chemical Engineering, University of Birmingham, Edgbaston B15 2TT, United Kingdom; b University Hospital Birmingham Foundation Trust, Burns Centre, Mindelsohn Way, B15 2TH Birmingham, United Kingdom

**Keywords:** Cell transplantation, Burn injury, Wound healing, Hydrogels, Spray application

## Abstract

This review gives an updated overview on keratinocyte transplantation in burn wounds concentrating on application methods and future therapeutic cell delivery options with a special interest in hydrogels and spray devices for cell delivery.

To achieve faster re-epithelialisation of burn wounds, the original autologous keratinocyte culture and transplantation technique was introduced over 3 decades ago. Application types of keratinocytes transplantation have improved from cell sheets to single-cell solutions delivered with a spray system. However, further enhancement of cell culture, cell viability and function in vivo, cell carrier and cell delivery systems remain themes of interest.

Hydrogels such as chitosan, alginate, fibrin and collagen are frequently used in burn wound care and have advantageous characteristics as cell carriers.

Future approaches of keratinocyte transplantation involve spray devices, but optimisation of application technique and carrier type is necessary.

## Graphical abstract

**Figure:**
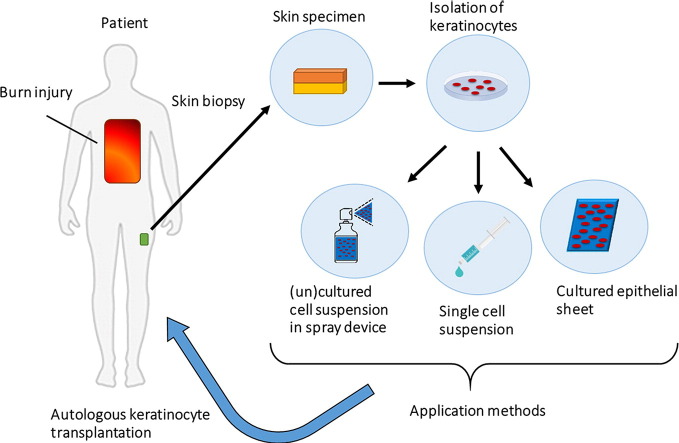

## 1 Introduction

Burn injuries are complicated wounds to manage with a relative high mortality rate in especially large area burns and elderly patients [1]. Substantial tissue damage and extensive fluid loss can cause impaired vital functions of the skin. Rapid epithelialisation is mandatory to restore the barrier function of the skin and enhance healing. Pathological scar formation (hypertrophic scarring) can occur as a long term sequelae of delayed wound healing. When healing is delayed, the potential short term common complications include wound infection affecting the local healing process or systemic inflammatory and immunological responses which subsequently can cause life threatening sepsis and multi-organ failure. In the United states, approximately 400,000 fire/burn injuries were recorded in 2014 in a population of about 300 million, including a total of 3196 (0.78%) fatal injuries (data from CDC in WISQARS Injury Mortality Report) [2].

Fortunately, survival rates have improved drastically over the last century due to advancements in burn care such as early surgical intervention, critical care support and wound care [3], [4]. However, despite further technological advancements in the last 30 years, survival rates have not improved significantly over the last three decades and now seem to be plateauing in countries with high-standard burn care [5], [6], [7].

Furthermore, since modern standard burn care allows the majority of patients to survive thermal injury, other outcome measurements aiming to improve quality of life become more relevant. For example, shortening length of hospital stay, decreasing the number of trips to the operating theatre and optimizing the quality of restored tissue. Functional and aesthetic outcome of the restored tissue are reflected by scar quality in terms of pigmentation, pliability, sensation, hair growth and function (prevention of scar contraction).

All of these factors require a specialized approach aiming on regeneration of tissue instead of tissue repair. Progress in short term results (lifesaving wound coverage) remains essential. Subsequently, advances of long term results are desired to facilitate the need for quality of life improvement of the increasing population of burn survivors. Answers to these challenges are sought in the field of tissue engineering. Although, advances in engineered skin equivalents and cell-delivery to the wound bed are emerging in burn care, they currently do not meet the expected results and translation to clinical practice is challenging. Keratinocyte delivery was the first skin cell transplantation successfully translated to the clinical burn care. In the last four decades this method has been investigated widely and numerous researchers have contributed to a variety of improvements. This review gives an updated overview on applications of keratinocyte delivery in burns and wound healing and future therapeutic cell delivery options with a special interest in hydrogels and spray devices for cell delivery.

## 2 Skin

### 2.1 Epidermis

**Fig. 1:**
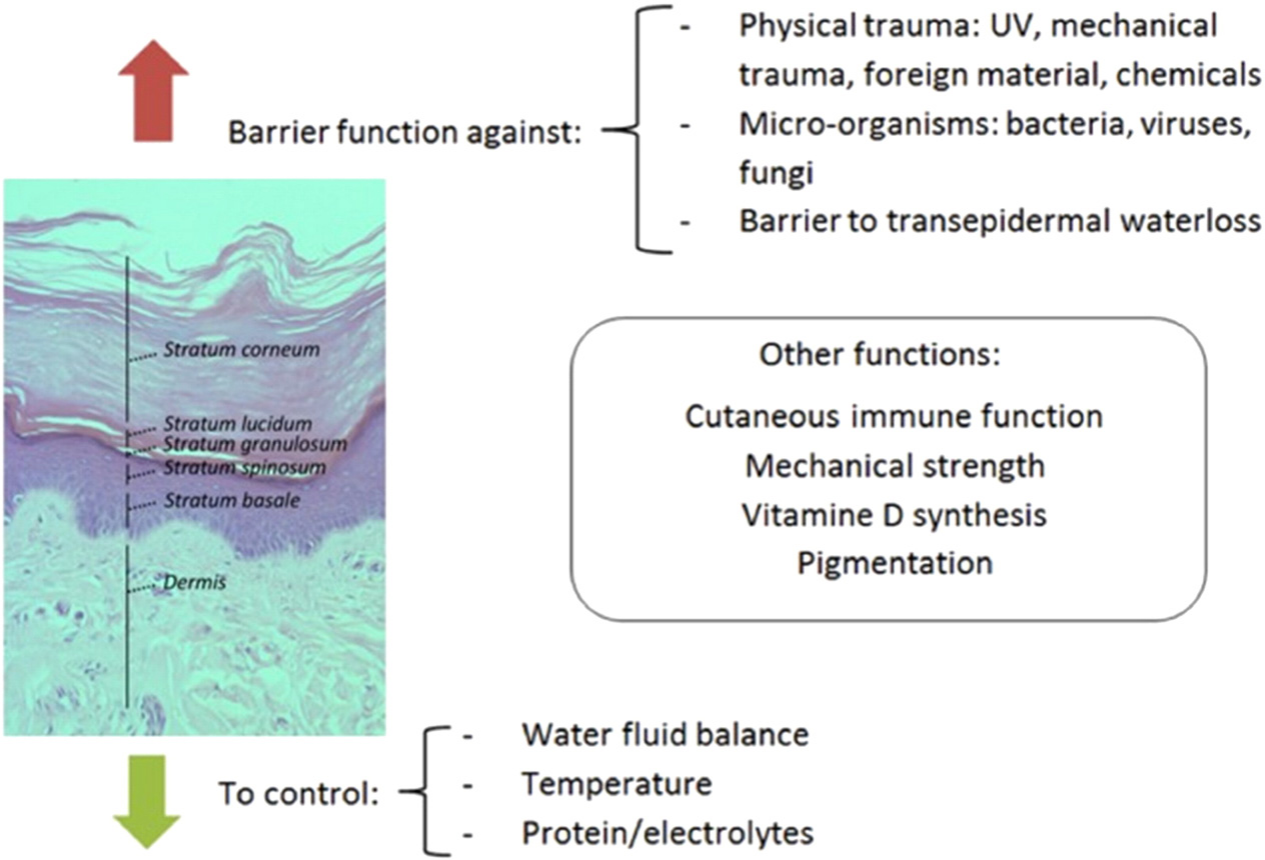
Layers and function of the skin. The uppermost layer of the skin is the epidermis. The epidermis consists of 5 main layers described from deep to superficial: stratum basale, stratum spinosum, stratum granulosum, stratum lucidum and stratum corneum. The epidermis has two distinct functions: a protective barrier function against trauma and fighting off pathogens as well as a controlling function regulating body temperature, fluid and electrolyte balance. Other functions of the epidermis include production of vitamin D, pigmentation, providing mechanical strength and it has a role in cutaneous immune function.

**Fig. 2:**
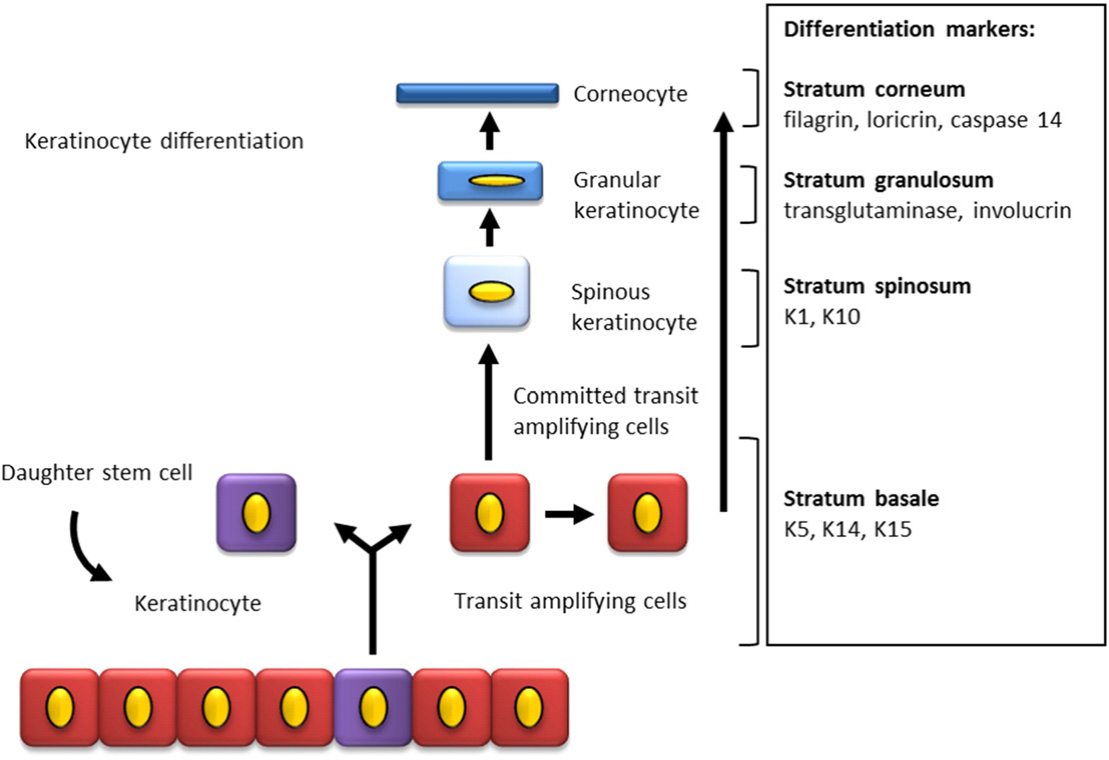
Keratinocyte differentiation and markers. Diagram is showing differentiation of keratinocytes in the epidermis with expression of stratification markers. Basal keratinocytes express Keratin 5, keratin 14 and keratin 15. When keratinocytes differentiate they move upwards into the suprabasal layers: stratum spinosum, stratum granulosum and finally stratum corneum. Differentiating keratinocytes express specific markers in each epidermal layer.

The skin is the largest organ of the body and has a barrier function, preventing the passage of water, electrolytes and pathogens (Fig. 1). The epidermis is predominantly formed from highly specialized epithelial cells called keratinocytes. Other cells which can be found in the epidermis include Langerhans' cells, melanocytes and Merkel cells, which are responsible for immune regulation, pigmentation and sensory function. Keratinocytes play a key role in epidermal restoration following injury through proliferation and re-epithelialisation (Fig. 2). Solely epidermal injuries will achieve re-epithelialisation from proliferated keratinocytes and heal by regeneration without scarring [8], [9]. Differentiated keratinocytes perform their barrier function through the provision of a mechanical barrier in the formation of a keratinised layer and by reacting to invasion of pathogens via release of pro-inflammatory mediators which subsequently attract leukocytes to the site of invasion.

[Source: histology image provided by Dr. G M Reynolds PhD CSci FIBMS, Liver Unit of Queen Elizabeth Hospital Birmingham, UK. Image adapted by Britt ter Horst, with permission from G M Reynolds.]

[Source: Britt ter Horst].

#### 2.1.1 Keratinocyte differentiation and proliferation markers

Keratinocytes proliferate from the basal cells of the innermost layer of the skin (*stratum basale*). The epidermal stem cells are attached by hemi-desmosomes to the *stratum basale* and can divide into either more stem cells, which persist indefinitely and to maintain the layer's regenerative capacity, or into transit amplifying cells which have limited division potential. As the transit amplifying cells continue to divide and proliferate, differentiation occurs. Throughout this differentiation process, the keratinocytes migrate upwards towards the *stratum spinosum* and *stratum granulosum* to eventually become corneocytes which form a relatively impermeable outer layer, the *stratum corneum*. Once fully differentiated, these corneocytes lose their nucleus and cytoplasmic organelles and will eventually be shed off via desquamation. The estimated time for turnover from epidermal stem cell to desquamation in healthy human skin is around 39 days [10].

During this process, keratinocytes express several differentiation proteins including keratins which are intermediate filament proteins in epithelial cells. Keratins play a host of important function including the provision of structural support, protection of epithelial cells from mechanical and non-mechanical stress and the regulation of apoptosis and protein synthesis [11]. There are 37 known functional human epithelial keratin genes, divided in type 1 and 2 genes. Mutations in these genes are associated with skin diseases such as epidermolysis bullosa simplex (keratin 5, 14) with structural weak epidermal basal cells or epidermolytic hyperkeratosis (keratin 1 and 10) [12]. Keratin expression is frequently used as a marker for epidermal proliferation and differentiation in cell culture, with keratin 14 (K14) being used for the basal layer and keratin 10 for the spinous layer. Other differentiation markers starting at the basal layer are K5, and K15, spinous layers K1 and K10, transglutaminase and involucrin, at the granular layer. Filagrine, loricrin and caspase-14 activation are hypothesised to play a role in terminal keratinocyte differentiation [13], [14], [15]. (Fig. 2).

#### 2.1.2 Factors promoting keratinocyte differentiation

A major regulator of keratinocyte differentiation is the calcium gradient. Extracellular calcium concentration is lowest in the stratum basale and gradually increases until the stratum granulosum. Elevated levels of extracellular calcium concentrations stimulate formation of intercellular contacts and the increase of intracellular free calcium concentrations via transmembrane calcium influx, which subsequently initiates differentiation via stimulation of the calcium receptor (CaR) [14]. This has consequences for the culture technique of keratinocytes in vivo, high calcium concentration induces differentiation, whereas in low calcium concentration keratinocytes remain proliferative [14], [15], [16].

E-cadherin provides adherens junctions for adhesion between cells which is crucial for keratinocyte differentiation. In addition, following a signalling pathway e-cadherin can increase the intracellular calcium concentration [14]. Furthermore, 1,25-Dihydroxyvitamin D3 (Vitamin D3) is known to influence keratinocyte differentiation by regulating gene expression and modulating calcium concentrations [17], [18].

Logically, factors that promote proliferation will inhibit differentiation of keratinocytes. Factors known to promote proliferation are TGF-α, vitamin A, transcription factor p63 and epidermal growth factor (EGF).

#### 2.1.3 Keratinocyte interaction with other epidermal cells

Within the epidermis, keratinocytes interact with other surrounding cell types for example, melanocytes. Melanin production (melanogenesis), occurs in the melanocytes and protects the DNA of melanocytes and keratinocytes from ultraviolet radiation and contributes to the colouration of the skin. Keratinocytes take up melanin via the melanin containing melanosomes produced by melanocytes [19].

The interactions between keratinocytes and fibroblasts in wound healing have been well described in literature, where a double paracrine signalling concept is proposed. Keratinocytes instruct fibroblasts to produce growth factors and cytokines such as keratinocyte growth factor, fibroblast growth factor-7, GM-CSF and IL-6 [20]. Consequently, expression of these growth factors initiates keratinocyte proliferation. The transcription factor activator protein-1 seems to play an important role in this process [21]. Furthermore, under the control of keratinocytes, fibroblasts can obtain a myofibroblast phenotype, which is important for wound contraction [20].

### 2.2 Dermis and basement membrane

Underneath the epidermis, the dermal layer acts a support network, providing strength and elasticity to the skin. Fibroblasts are the key cells of the dermis. Fibroblasts are responsible for the production and maintenance of the extracellular matrix which is formed by fibrous components (collagen and elastin) embedded in non-fibrous elements such as proteoglycans and glycosaminoglycans (GAGs). Collagens are the main structural element of the extracellular matrix (ECM) and provide tensile strength, regulate cell adhesion and support migration. Other cellular components include endothelial cells, smooth muscle cells and mast cells [22]. The vascular deep and superficial plexus lie within the upper and lower part of the reticular dermis respectively and supply the dermis and epidermis. The epidermis and dermis are firmly connected by the basement membrane, and the epidermal-dermal junction is bordered and stabilized by the anchoring of keratinocyte-derived collagen (type VII) fibrils into the dermis. Additionally, collagen XVII, a structural component of hemidesmosomes, mediates the anchoring of basal epithelial cells to the basement membrane [23]. If this junction is disrupted, serious morbidity such as seen in epidermolysis bullosa can occur [12].

Several structures originate in the dermis and extend into the epidermis such as sensory nerves, sweat glands and hair follicles. Hair follicles are lined with epidermal keratinocytes and contain multipotent stem cells [24]. Therefore, if the dermis is only partially injured these adnexal structures can deliver cells that can proliferate, migrate and regenerate the epidermis. However, the dermis lacks the intrinsic capability of regeneration and heals by fibrosis and scar formation. Sensory nerves are responsible for mediating pain and itch, control inflammation and there is evidence that shows that they may also influence the remodeling phase [9]. After skin injury the body starts a remarkable healing process which often results in complete regeneration.

## 3 Wound healing and keratinocytes

### 3.1 The role of keratinocytes in wound healing

The skin barrier function can be disrupted by trauma such as a thermal injury. Wound healing usually occurs via four overlapping phases; haemostasis, inflammation, proliferation and remodeling. Normally this process is sufficient to allow the skin to repair itself after injury. However, extensive skin loss, as seen in burn victims, requires intervention to allow for tissue restoration. Burn injuries are often caused by heat, however, electricity, radiation, chemicals or friction can also result in similar injuries clinically [25]. Following thermal injury, a complex healing process will start with the involvement of numerous specialized and interacting cells, molecules and pathways. The cellular response involves macrophages, platelets, fibroblasts, epithelial and endothelial cells. In addition to the various cellular interactions, proteins and glycoproteins such as growth factors, cytokines, chemokines, inhibitors and their receptors can also influence healing. Although, burns heal differently from normal wound healing, the phases of healing remain the same [26]. Keratinocytes and fibroblasts play an important role in the proliferative phase which is focused on the replacement of the damaged ECM and restoration of tissue structure and function. Activation of keratinocytes and fibroblasts by macrophages via cytokine and growth factor release causes angiogenesis, collagen production, ECM production and epithelialisation [27].

#### 3.1.1 Angiogenesis

The restoration of the vascular network is essential as angiogenesis supports cell activity by providing oxygen and nutrients to the wound bed. Once endothelial cells are activated by macrophages, they loosen their cell to cell junctions in order to migrate. This process as well as endothelial proliferation is encouraged by a hypoxic and acidotic environment which is typically found in wounds. Finally, revascularisation occurs when sprouted vessels organise into capillary networks. Vascularisation consequently neutralizes the hypoxic and acidotic wound environment and leads to decreased production of angiogenic factors. This eventually results in reduction of endothelial cell migration and proliferation [8], [28].

#### 3.1.2 Epithelialisation

Within hours of injury re-epithelialisation starts with a vital role being played by keratinocytes. The quantity of epidermal stem cells residing in stem cell niches such as in the hair follicles, sebaceous glands and basal layers of the interfollicular epidermis determines the regenerative capability of the skin [8], [24].

**Fig. 3:**
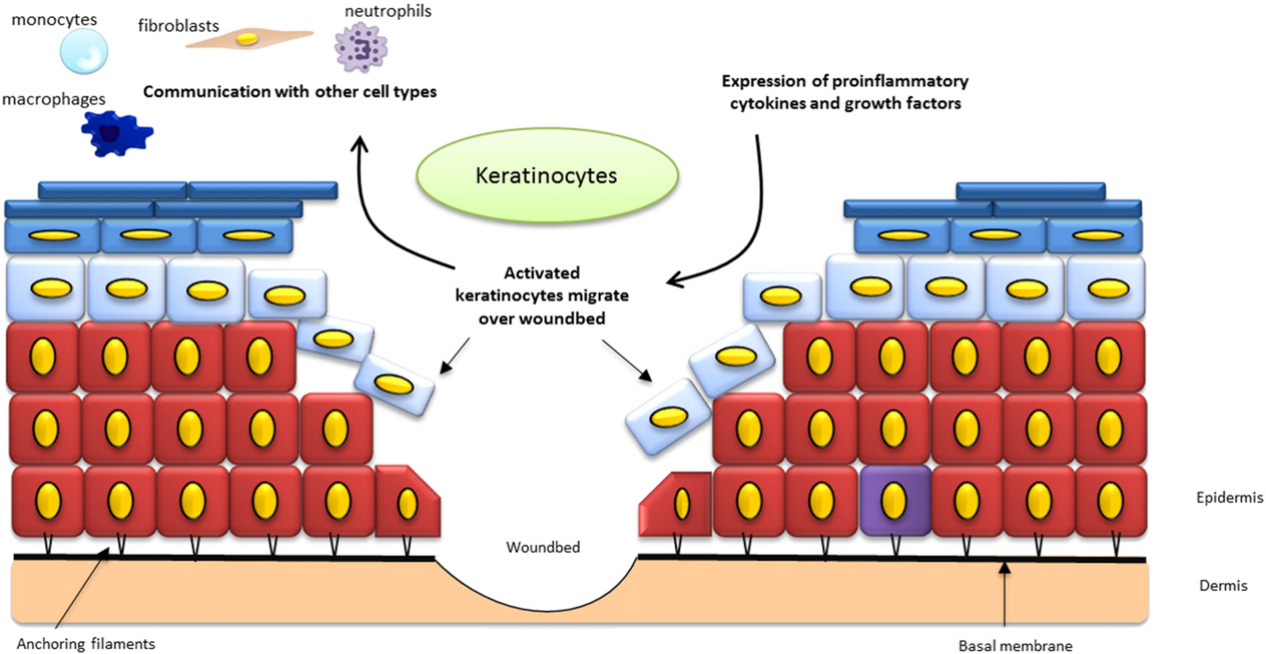
Role of keratinocytes in re-epithelialisation. Schematic illustration of a skin injury with keratinocytes as key cells. Keratinocytes are activated via pro-inflammatory cytokines and growth factors released in the wound bed. Once activated, keratinocytes from the wound edges and dermal appendages migrate over the provisional matrix and finally close the defect in a process called epithelization. When the basal layer is spared from injury, basal keratinocytes can support this process by upward migration as occurs in non-injured skin. Activated keratinocytes communicate with other cell types present in the epidermis. Epithelial cells proliferate and differentiate to achieve a stratified epithelium with restoration of the barrier function of the skin. Maturation of the wound continues over a period of several months with fibroblasts remodeling the underlying dermis.

Activated by growth factors released by macrophages, keratinocytes migrate to the wound bed and fill the defect (Fig. 3). In order for keratinocytes to start their migration they undergo phenotypical alterations by loosening of intercellular adhesions, although some desmosome contacts are sustained [8]. Furthermore, cells can separate from the basal layer once hemidesmosomes are disrupted which allows them to migrate laterally [8], [29]. When integrin receptors are expressed, the keratinocytes flatten and the altered basal keratinocytes migrate over the granulation tissue to form a monolayer of epithelial cells, but remain under the non-viable eschar of the burn wound. While moving they secrete proteolytic enzymes that enable the degradation of provisional matrix and promotes further cell migration [30]. After a confluent sheet of cells covers the wound bed, the cells then divide to form a multi-layered stratified epithelium and mature under the influence of TGF-β1 and TGF- β2 [31].

[Source: Britt ter Horst].

Keratinocytes play a vital role in especially the proliferative phase of burn wound healing leading to epithelialisation and restoration of the vascular network. For this reason and the possibility of in vitro keratinocyte culture, keratinocytes are considered an excellent candidate for cell transplantation.

### 3.2 Pathophysiology of burn injury

Major burn injury, defined as approximately 20% of the total body surface area (TBSA) burned, causes burn shock due to severe haemodynamic and haematopoietic dysfunction [26] secondary to immediate evaporative and direct fluid losses [32], extensive loss of proteins, reduced colloid oncotic pressure and wound oedema development. Without intervention the fluid loss will result in desiccation of deeper tissues and further cell death leading to an increase in wound depth. A moist wound bed is necessary for epithelial cell movement and therefore for successful re-epithelialisation [9]. Furthermore, with disruption of the barrier function of the skin, microorganisms have easy access and when entering the microcirculation can cause systemic infection. Presence of these acute systemic responses are related with increased morbidity and mortality numbers [33], [34], especially in the elderly [1].

### 3.3 Rational for keratinocyte transplantation

Traditional therapy for severe burns is surgical debridement and autologous skin graft. However, with extensive burn injury healthy donor site is scarce and alternatives to restore skin function are necessary. When rapid epithelialisation can be achieved the skin barrier function is restored and this can determine a patient's likelihood of survival. Clearly, it is important in the treatment of a burn injury to focus on quick re-epithelialisation. Therefore, development of successful and efficient autologous skin replacement techniques is highly desirable. Wound closure will not occur without epithelialisation and epithelialisation will not occur without the presence of keratinocytes in the wound bed [8]. To achieve faster re-epithelialisation, keratinocyte transplantation was introduced as part of the burn wound care arsenal over 30 years ago. However, the original autologous keratinocyte transplantation technique has several disadvantages which has spurred researchers to seek for improvements in cell culture technique, delivery systems and also the optimisation of the timing of keratinocyte transplantation [35].

## 4 Cell transplantation technology

### 4.1 Cell source

Keratinocytes and their progenitor cells can be sourced locally from stem cell niches in hair follicles. Several stem cell niches are known: mesenchymal stem cells (MSC) resident in the dermal papilla and multipotent hair follicle stem cells (HFSC) and melanocyte stem cells in the superior bulge [36]. HFSCs are essential for normal morphogenesis of hair follicles, sebaceous glands and contribute to formation of the three epithelial cell lines [37]. Progenitor cells also reside in the bone marrow and could arise from embryonic cell lines [38].

### 4.2 Methods of human keratinocyte cell culture

#### 4.2.1 Historical development of keratinocyte culture

The first successful in vitro human keratinocyte expansion, achieved by Rheinwald and Green in 1975, paved the way towards autologous cell transplantation in burn care [39]. Keratinocytes were successfully cultured in the presence of fibroblast feeder cells. However, to prevent these fibroblasts from outgrowing the keratinocyte population, irradiated murine 3T3 fibroblast feeder cells, which have lost their mitotic ability but remain metabolically active, were used [39]. Besides the use of feeder cells, culture media often contains fetal calf serum with growth factors, hormones and antibiotics [39], [40], [41]. Keratinocytes are able to be grown into colonies and subsequently form a stratified epithelium and human keratinocyte stem cells were proven to have an enormous proliferation potential. Subsequently, small cell sheets of two or three layers of confluent keratinocytes were produced and not long after, the first human transplantation became a reality [42], [43].

Keratinocytes cultured for clinical use must have the regenerative capability to form an effective epidermis after transplantation. It is thought that in vitro differentiating keratinocytes do not contribute significantly to regeneration in vivo [44]. Instead, keratinocyte stem cells and their transient amplifying cells seem to have excellent regenerative capacities [45]. Thus, culturing keratinocytes that keep the ability to produce progeny once transplanted, seems key for successful epidermis formation following transplantation.

The degree of differentiation in vitro can be controlled by the method of culture. Fully differentiated keratinocytes (confluent) multilayers as well as pre-confluent single cells can be produced and delivered to the wound bed [16], [46]. In a histological comparison of cultured pre-confluent and uncultured keratinocytes seeded on a collagen-GAG matrix in a pig model, both provided a fully differentiated epidermis in 14 days. A thicker and confluent cell layer, however, was obtained more rapidly with the cultured cells [47]. The original culture method has been the subject of much debate, because the murine fibroblast feeder layers can potentially result in transplantation of animal components with the keratinocyte product. Due to the serious risk of animal-derived disease transmission to human epithelium in the transplantation process, it is not advised to use undefined xenogeneic materials in the treatment of patients. Additionally, radioactive irradiation of murine fibroblasts in this technique is accompanied with higher costs and potential uptake of irradiated DNA via the murine fibroblasts into the transplanted keratinocytes might cause cell destruction [48].

#### 4.2.2 Progress towards xenobiotic free culture techniques

To limit or exclude the transmission risk, other keratinocyte culture protocols without feeder cells and limited or no use of xenogeneic media products have been developed [16], [49]. Jubin et al. showed that human keratinocytes can be successfully expanded in co-culture with non-irradiated autologous human fibroblasts in Rheinwald and Green but still required medium supplemented with fetal bovine serum (FBS) to maintain their proliferative phenotype in vitro [50]. A further approach to minimise xenogeneic products in culture media was introduced when serum free medium was used for the expansion of keratinocytes with non-irradiated human fibroblasts on several substrates [49]. However, FBS still had to be used to expand the human fibroblasts initially.

Although the culture media are free of serum, often other products used in the culture media still contain animal-derived proteins. This could be solved by the use of only human material, however the risk of infection remains and high costs in an already expensive process makes this option less favourable. Successful culture of human keratinocytes in a serum-free and feeder-free culture was demonstrated in vitro in a skin equivalent model by Coolen et al. With the addition of collagen type IV, serum substitute and keratinocyte growth factor (KGF) a differentiated epidermis could be formed [51]. Lamb et al. demonstrated that although keratinocytes grown in serum-free and feeder-free conditions did show sufficient propagation, these cells were not able to support mature epidermis formation in an in vitro skin model. However, when re-introduced to a serum-containing media they then did form a stratified epidermis. Moreover, when heat-inactivated serum was used an improved stratified epidermis was formed, indicating that serum-products also contains (heat-sensitive) factors that can inhibit in vitro epidermis formation [52].

Lenihan et al. compared three commercially available feeder-free media systems; CnT-07 medium (CellnTech, Bern, Switzerland), EDGS (Gibco), S7 (Gibco) with the original Rheinwald and Green method [39] for expansion of human keratinocytes for clinical usage. A maximum of 3 weeks culture time (passaged twice) was allowed, as the ideal transplantation window was considered between 4 and 20 days. They found that all three feeder free culture media supported keratinocyte growth. However, the only fully xenobiotic free media (S7) had a low cumulative population doubling time and therefore did not reach sufficient cell numbers to be considered for clinical usage at day 21 and was therefore excluded from further analyses [53].

Using feeder free and serum free media is less labour intensive and beneficial for use in the clinical setting, further research will have to show whether keratinocytes maintain their proliferative potential in vivo. Besides elimination of xenobiotic materials from culture media, the elimination of antibiotics is a further goal to improve keratinocyte cell expansion for usage in the clinical setting [54].

#### 4.2.3 Additions to keratinocyte culture

To further encourage and optimise skin regeneration following burn injury, improvements have been made to the keratinocyte transplantation process. These have included the addition of multiple other cell types and growth factors during keratinocyte culture or transplantation.

The addition of melanocytes in the keratinocyte culturing process has been proposed to solve the problem of potential irregular pigmentation of the post burn scar. Co-culturing of keratinocytes with melanocytes has been investigated in patients with vitiligo and in full thickness wound healing in animal models [55], [56], [57], [58]. In humans, more evidence is available for uncultured cell suspensions containing keratinocytes and melanocytes for the treatment of hypopigmented lesions [59], [60], [61], [62]. However, these pilot studies are limited by small sample sizes and lack of controls. Controlled clinical studies are needed to support the findings before this technique can be accepted as standard clinical practice.

Cultured epithelial autografts lack a vascular plexus and burn wounds often have insufficient vascularisation due to the disruption of the dermal layer. Therefore, approaches to promote angiogenesis via the addition of autologous or allogenic endothelial cells into skin grafts have been proposed [63], [64]. Also, adipose derived stem cells (ASCs) have received attention with respect to their potential to enhance wound healing. Huang et al. seeded human ASCs onto a dermal acellular skin substitute in vitro to enhance vascularisation. When transplanted to full thickness wounds in nude mice, an increase in blood vessel density was found two weeks post transplantation compared to controls [65]. Another approach is to add growth factors like epidermal growth factor (EGF) [66]. Additionally, co-delivery of cultured keratinocytes with EGF in a fibrin matrix demonstrated improvement of epidermal regeneration in full thickness wounds in a murine model [67]. Furthermore, Supp et al. genetically modified keratinocytes with an overexpression of vascular endothelial growth factor (VEGF) to stimulate angiogenesis in skin substitutes in animal studies [68], [69]. Besides adding factors, modifications of the 3D structure of skin substitutes to stimulate faster ingrowth of vascular structures have also been proposed [70]. Of interest from a tissue engineering perspective, is whether transplanted cells actually survive and function in vivo.

### 4.3 Keratinocyte viability after transplantation

Cell survival of transplanted keratinocytes in vivo is of great interest for tissue engineering purposes. Vernez et al. evaluated the cell viability and apoptosis balance in clinical samples taken from cultured epidermal autografts prior to transplantation [71]. Although, all samples showed high levels of cell viability and low levels of apoptosis, variable biological activity of certain parameters between samples of different patients was observed. It was suggested that this could impact on therapeutic efficacy [71]. In a pig model Navarro et al. found no altered cell viability before and after spraying a suspension of cultured keratinocytes to full thickness wounds [72]. Duncan et al. examined cultured human keratinocyte proliferation measured with the MTT assay after spray delivery to a de-epidermalised dermis (DED) in vitro and found no significant cell death or reduced cell proliferation [73]. These studies seem to support that cells remain viable and maintain proliferative capability after spray cell delivery to a wound bed.

## 5 Application types of keratinocytes transplantation

### 5.1 Introduction: grafting of burn wounds

Ambroise Paré (1510–1590 CE) was probably the first to describe the surgical intervention for early excision of burn wounds [74]. Surgical burn care has progressed tremendously since then and methods which have now become well established in burn care include early excision of burn wounds, the development of autologous and allogenic skin grafts and the use of skin substitutes [75].

Techniques involving transplantation of healthy human skin to damaged areas are still the gold standard in deep or full thickness burn wounds. However, challenges arise when large areas are affected and donor sites are scarce. Subsequently, many skin graft expansion techniques have been developed to reduce donor site size, these techniques include meshing of the graft (maximum expansion ratio of 1:9), modified meek technique (expansion ratio of 1:9), epidermal blister grafting (expansion ratio of 1:1) and several techniques of micro grafting such as epidermal CelluTome™ micro-grafting (expansion ratio: 1:6), or Xpansion® micro-grafting (maximum expansion ratio: 1:100). Developers of cellular based techniques claim to deliver even higher expansion rates, such as cultured epithelial sheets (expansion rate 1:1000) and uncultured cell suspensions (maximum expansion rate 1:100) [76], [77]. However, to the knowledge of the authors no other studies have been able to support these findings.

Furthermore, procedures to harvest skin are time consuming, can lead to longer healing times with prolonged hospital stay and can be accompanied by donor site complications. Additionally, skin grafts do not always meet the desired cosmetic outcomes.

Therefore, methods to enhance the results of skin grafting and alternatives to it have been subject of much research in the last few decades. Specifically, progress towards a permanent epidermal replacement or its improved regeneration is the main goal of cellular based therapy. In Fig. 6 different methods of autologous keratinocyte transplantation are schematically summarized.

**Fig. 4:**
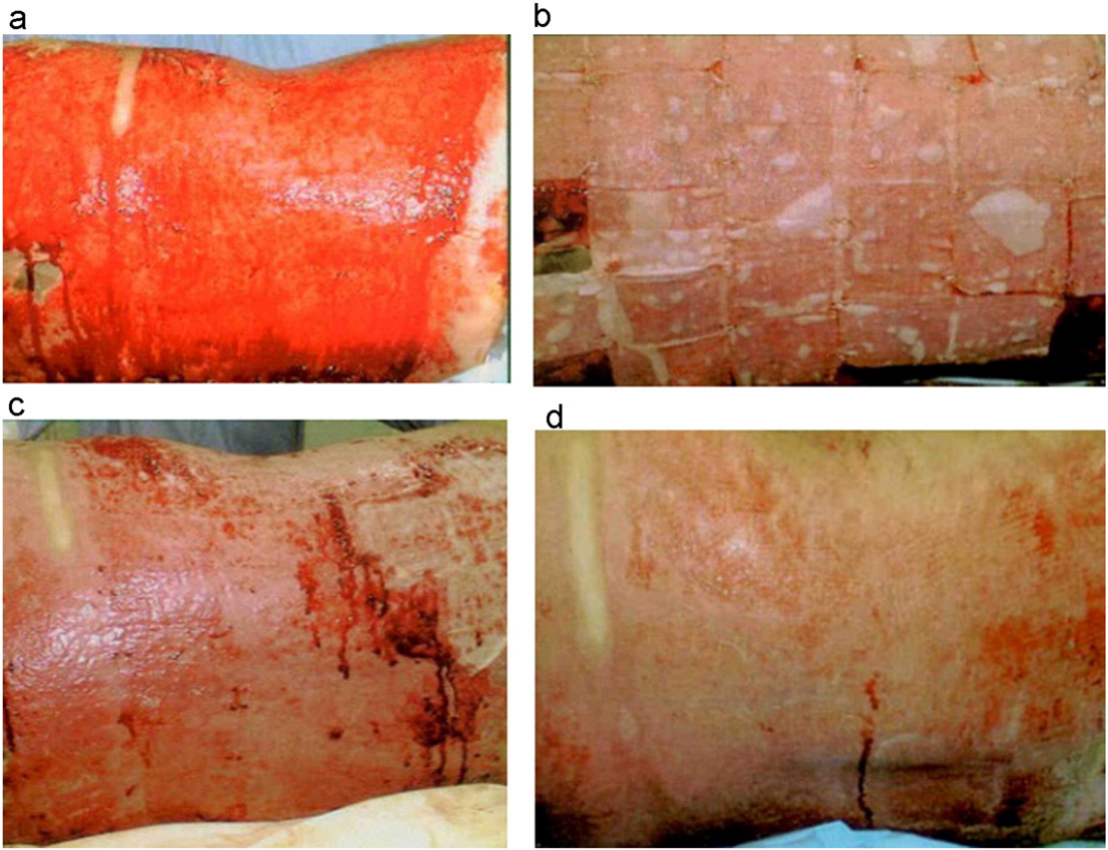
Burn wound coverage with cultured epithelial autografts applied in sheets. In this example, successful burn wound healing in about 2 weeks was achieved when the sheets were removed a week after application. a) Deep second degree burn in the back of a 29-year old patient after excision of the burn b) application of cultured keratinocyte sheets c) removal of sheets 8 days after surgery and d) complete healing 16 days after surgery.

[Reprinted from Burns Volume 41, Issue 1, Pages 71–79, Cultured autologous keratinocytes in the treatment of large and deep burns: A retrospective study over 15 years, Celine Auxenfans, Veronique Menet, Zulma Catherine, Hristo Shipkov, Pierre Lacroix, Marc Bertin-Maghit, Odile Damour, Fabienne Braye, Copyright (2017), with permission from Elsevier.]

**Fig. 5:**
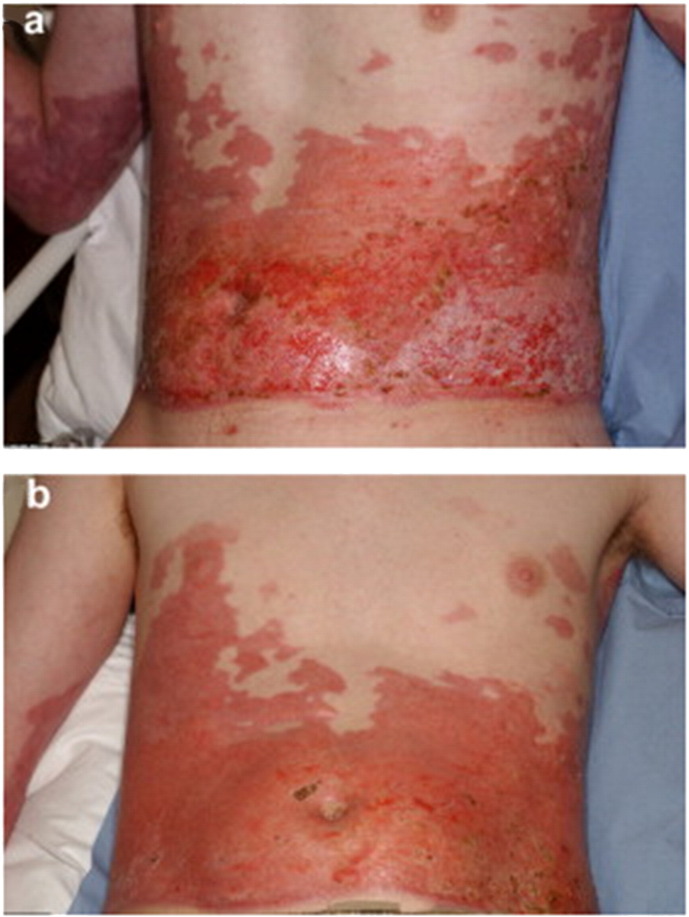
Spray delivery of cultured keratinocytes to enhance burn wound healing. In this example, a mixed depth burn to the abdomen was treated with solely sprayed cultured keratinocytes (no additional mesh grafting) 27 days after injury. The wound was considered to have healed completely 10 days after treatment. Unfortunately, long term outcomes in terms of scar quality were not available for this patient.

[Reprinted from Burns Volume 36, Issue 3, Pages e10–e20, Sprayed cultured autologous keratinocytes used alone or in combination with meshed autografts to accelerate wound closure in difficult-to-heal burns patients, S. Elizabeth James, Simon Booth, Baljit Dheansa, Dawn J. Mann, Michael J. Reid, Rostislav V. Shevchenko, Philip M. Gilbert, Copyright (2017), with permission from Elsevier.]

**Fig. 6:**
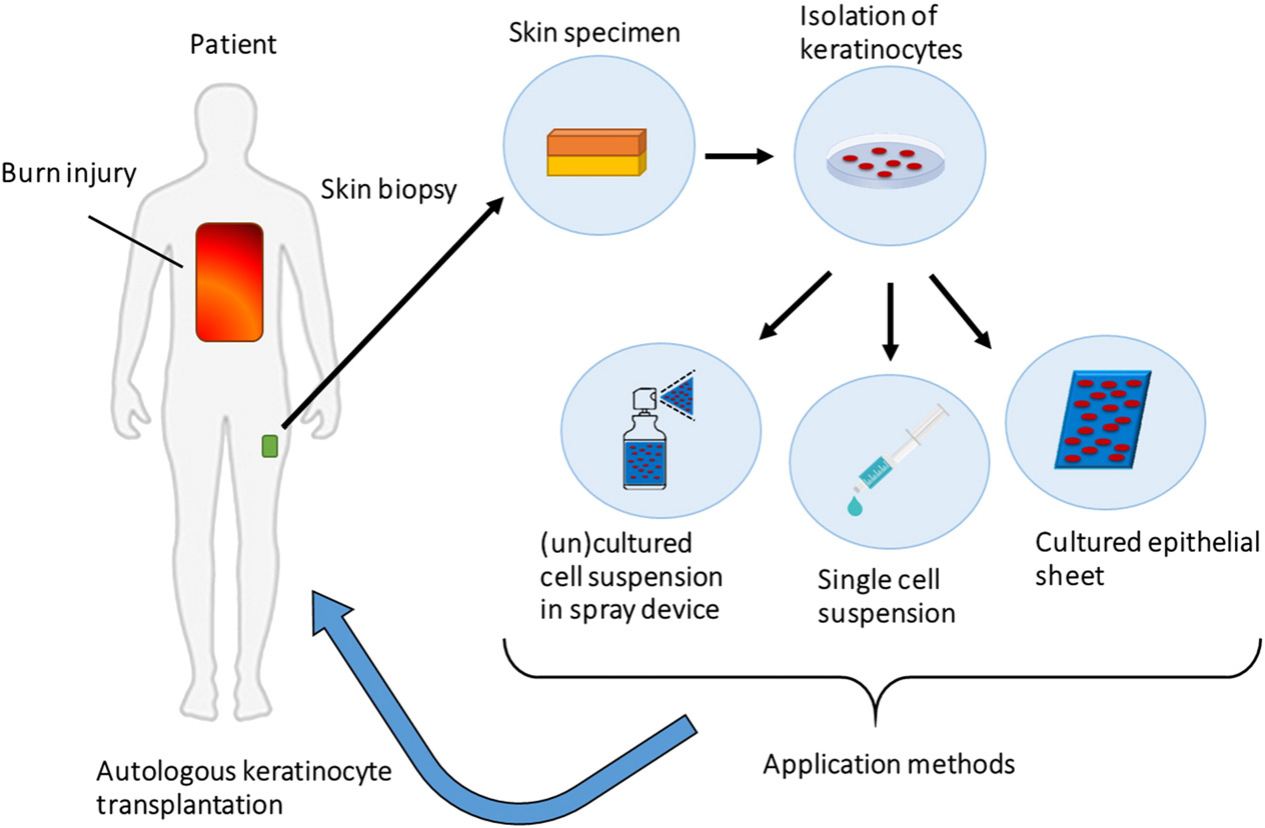
Methods of autologous keratinocyte transplantation to burn wounds. In patients with burn injury keratinocytes can be isolated from a small skin biopsy as illustrated above. The autologous keratinocytes can be cultured and delivered to the wound bed of the patient by several methods. First to be developed was a sheet of cultured epithelial cells, thereafter a single cell suspension applied to the wound by dripping from a syringe and latest development is application of cultured or uncultured cells in single-suspension with a spray device.

[Source: Britt ter Horst].

### 5.2 Cultured keratinocyte sheets

#### 5.2.1 Cultured autologous keratinocyte sheets

In 1981, O'Connor et al. reported the first transplant of cultured autologous keratinocytes to treat a burn injury [43]. Cultured epithelial autografts (CEA) were developed to replace the epidermis and restore the barrier function of the skin [78], [79]. In the last three decades CEAs have been adapted and introduced to the clinical setting (Fig. 4).

Nowadays, several commercialised bioengineered skin products derived from autologous cells are available. In general, clinicians harvest autologous skin and the company produces a graftable substrate seeded with the autologous cells for clinical use in approximately 2 weeks (Epicel, Genzyme, Cambridge, MA and Laserskin, Fidia, Italy). The timeframe wherein viability of the grafts can be ascertained (shelf-life) is 24–48 h. These services will often involve high costs and a certain waiting time and narrow application timeframe.

In 2007, the FDA approved the use of CEAs for use in patients with deep dermal or full thickness burns greater than, or equal to 30% TBSA (Epicel, Genzyme, Cambridge, MA) [43], [80], [81]. The main advantage of cultured epithelial autografts is that large areas of the body can be covered with autologous cells derived from a small biopsy and improvement in the speed of re-epithelialisation has been reported. In terms of cosmetic results, CEA seems to have better results when compared to wide mesh autograft in extensive burns [82]. However, several authors who have reviewed the use of cultured epithelial autografts in burn care have found variability in terms of graft take and cosmetic outcomes [46], [83], [84].

A major disadvantage of this technique is the long time-interval between biopsy and grafting. Although the average culture time has improved from 5 [43] to about 3 weeks [85], [86], variability among patients has been described, especially among different age groups [87]. Following burn excision, the wound can be temporary covered with allograft and/or xenograft dressings for several weeks until CEA is ready. However, this is related to a higher risk of wound colonization and infection [46]. The ideal timing for keratinocyte transplantation is difficult to determine as it is dependent on several factors including hospital facilities and patient conditions [86], [88].

Furthermore, both short and long term clinical limitations such as the formation of bullae, poor take rates, fragility of the sheets and wound contractures have been reported [88], [89], [90]. These may be due to the lack of a dermal component that is necessary to support the new epidermal layer.

The restoration of the dermis is important for the skin to regain mechanical strength and to facilitate adherence of the new or transplanted epidermis [36]. Although in one study, an advanced application technique with allograft wound bed preparation and combination of CEA with wide meshed autograft seems to improve take rates up to 84% [91].

Cell culture is an expensive process and the cost/benefit relationship of this method is heavily debated [92]. Finally, the potential of graft site malignancy after keratinocyte transplantation has been highlighted [93], [94]. However, the type of malignancy reported, squamous cell carcinoma, is also known to occur in burn wounds and scars in the absence of keratinocyte transplantation [95].

#### 5.2.2 Introduction of dermal substitutes including cultured keratinocytes

With a complete absence of a dermal component, the cultured keratinocytes are thought to be of limited value in treating full-thickness burns due to the poor quality of the resulting epidermis. Consequently, this has led researchers to optimise the wound bed via the use of allogenic or artificial substitutes prior to keratinocyte transplantation. A further approach is to grow or seed the cultured keratinocytes on a (dermal) substitute to facilitate secure transplantation and improve healing potential [96]. This concept was introduced by Hansbrough and Boyce in 1989 [97]. Many types of delivery systems have since followed, and have been extensively discussed in the literature throughout the years [98], [99], [100].

Limitations in keratinocyte cell culture methods and transplantation have impeded the widespread use of this technique in the clinical setting. The use of single-cell suspension was introduced predominantly to shorten the culture time.

### 5.3 Autologous keratinocyte transplantation in suspension

To overcome the main negative features of epidermal sheets which are the long culture times and poor cell adhesion to the wound bed, delivery of cells in suspension form has been investigated.

While epidermal sheets contain cultured confluent cells that are passed the phase of exponential growth, cell suspension delivery systems can be designed to contain pre-confluent cells. Ideally, these cells are harvested or passaged when reaching a 70–80% coverage of culture dishes to ensure their proliferative capability and avoid confluence, hence the term pre- or sub confluent cells. When a sufficient cell number is reached (after approximately 2 weeks of culture), the cells are detached and suspended in a saline solution for clinical use. As differentiation in vitro is not desirable, keratinocytes in a pre-confluent suspension form is often preferred for transplantation (Fig. 5).

Nowadays, several commercially available spray cell delivery products are used clinically to enhance burn wound healing. These techniques can be categorised by the type and level of confluence of the transplanted cells.

#### 5.3.1 Pre-confluent keratinocytes suspension

The use of pre-confluent cells can shorten culturing time and facilitate more rapidly available cellular grafts, which in theory is likely to reduce the risk of wound infections and consequently the length of hospital stay [101], [102]. A commercial suspension consisting of autologous pre-confluent keratinocytes has been available since 2007 for aerosol delivery. Hartman et al. treated 19 patients with deep dermal face and neck burns with a spray apparatus with an estimated spray pressure of 8.2 mm Hg, which seems to be a surprisingly low delivery pressure using autologous cultured epithelial cells of 80–90% confluence at the end of passage 0 [103].

An alternative commercially available cell spray system is Keraheal^tm^, which was developed by MCTT, Korea. This system utilises an autologous non-differentiated pre-confluent keratinocyte suspension, which is sprayed to the burn wound followed by fibrin spray application. The Keraheal^tm^ methodology is similar to conventional CEA and requires 2–3 weeks of culture time, but the cells are provided in a suspension instead of sheet. To date, two single centre retrospective studies have evaluated the clinical outcomes of the sprayed cell suspension in combination with wide meshed skin grafts in a total of 39 (6 patients who died or were lost to follow up were excluded from follow up analyses) patients with severe burns. Graft take rate two weeks after application is surprisingly different between the studies, but after 8 weeks the take rates are both above 90%. A follow up of 1–2 years was achieved in both studies with a Vancouver scar scale (VSS) assessment. Scar evaluation 12 months after surgery was lower in Lee et al. with an average VSS of 3 compared to an average VSS of 5 in Yim et al. [101], [102].

Various developers have introduced adjustments to the technique in terms of the application device, cell detachment process, confluency of transplanted cell and application setting in order to meet clinical needs.

#### 5.3.2 Uncultured keratinocytes suspension

A further approach is the use of uncultured autologous cells for direct application onto burn wounds without pre-processing in a tissue culture lab. In a single procedure, a small piece (2 × 2 cm) of skin is harvested by the surgeon and then placed in an enzymatic solution followed by manual scraping of the epidermal layer, the skin specimen is placed in a buffer solution and subsequently filtered before use. The company provides a kit which allows the clinical team to process the cells in a single treatment session without the need for a lab technician or transport of the cells elsewhere [83], [104]. The use of an uncultured mixture of autologous epidermal cells (keratinocytes, melanocytes, dermal fibroblasts and Langerhans cells) was introduced to clinical practice in 2005 as a standardized spray device under the name ReCell (Avita Medical Europe Ltd., Melbourne, UK).

The purported benefits of this system are the elimination of lengthy culture times and the delivery of a mixture of autologous epidermal cells.

Since its introduction, several studies have demonstrated promising outcomes with the use of ReCell for acute burn wounds or in the treatment of hypopigmentation. These studies ranged from case reports to larger comparative studies [105], [60], [106], [107], [108], [109]. Although most papers have shown promising results, the potential value of spray cell transplantation in burns is difficult to evaluate due to the heterogeneity of the studies in terms of clinical outcomes explored, patient population, wound characteristics, type of treatment and study design [99]. Gerlach et al. used a similar approach with direct application of an uncultured autologous epidermal suspension on the wound bed using a fine needle spray in a single treatment session. Although, a small number of patients was treated and results were not compared to controls [110], [111]. The question arises whether a large wound area can be covered by the harvest of cells without expansion from a small skin specimen. In an in vitro study an expansion ratio of over 1:100 was calculated for uncultured cells sprayed with a density of 10^4^ cells/cm^2^ for an estimated surface coverage [112]. However, there is no other literature to support the claimed expansion rate.

To allow the comparison of clinical data, it has been recommended by the National Institute for Health and Clinical Excellence (NICE) that studies evaluating spray delivery of uncultured cells need to include at least: the time to 95% healing of the burn wound, length of hospital stay, scar assessment, physical function and cosmetic appearance of the burned area and compare these results with the current standard of care [69]. To date, randomized controlled clinical trials and non-commercial studies investigating effectiveness compared to conventional treatment are lacking. Challenges arise in consistent assessment for burn wound healing as objective non-invasive assessment tools have not yet been incorporated widely in routine burn care and might not be superior to visual expert assessment [26]. Therefore, researchers rely on subjective clinical assessments for acute burn wound healing and late outcomes in terms of scarring [113].

#### 5.3.3 Allogeneic neonatal keratinocytes suspension

Several research groups have explored the possibility of the transplantation of fetal allogenic cells with the purpose of stimulating regeneration of residual cells in the wound. Neonatal foreskin derived allogenic cells have low immunogenic properties which is preferred in tissue transplantation. This work has resulted in the creation of skin substitutes that have been seeded with allogenic cells such as Apligraf (Organogenesis, Canton, MA) and Orcel (Ortec International, Inc., New York, New York).

Similar to the developments in autologous cells delivery, allogenic cell suspensions have also been investigated as an alternative method of cell delivery. A cell suspension, code named HP802–47, which contains allogenic neonatal non-proliferating human keratinocytes and fibroblasts in thrombin was developed for the use in chronic wounds. Multi-centre randomized controlled phase IIa and IIb studies were conducted and have demonstrated promising outcomes in wound closure of venous leg ulcers [114], [115], [116], [117], [118]. Subsequently, a double blinded Phase III study followed in North America and Europe comparing wound closure after HP802-247 treatment or placebo in venous leg ulcers. However, the study was unexpectedly halted in the preliminary stages due to disappointing results [119]. To the knowledge of the authors, no clinical studies have been conducted for the treatment of burn wounds with HP802-247.

#### 5.3.4 Other clinical studies using cell sprays

Delivery of mesenchymal stem cell (MSC) to (burn) wounds is considered very promising due their capacity to differentiate into multiple lineages and potential beneficial effects on the immune response [54]. However, only few clinical studies investigated the use of mesenchymal stem cells to treat burn wounds have been performed so far [120], [121], [122]. Ueda conducted a pilot study of 10 patients treated with cultured epithelial autograft (CMEA) delivered to deep dermal burn wounds without adverse events and a healing time of approximately 12 days (range 7–14 days) [123].

Iman et al. compared spray delivery versus intradermal injection of autologous cultured keratinocyte-melanocyte suspension to treat hypopigmented burn scars in a total of 28 patients. Although patients might show a beneficial result with pigmentation, no statistical difference in type of application was found [59].

Cell transplantation techniques have changed significantly after the introduction of different cell-carriers and various forms of cell spray techniques. Nevertheless, some shortcomings of the suspension application technique have yet to be addressed.

For example, spraying on an uneven wound bed that often also occurs on a curved body contour, can result in uneven spreading of the of the cell suspension or dripping off the wound bed [46], [124]. A potentially useful development of keratinocyte transplantation is to improve the method of delivery in order to optimise cell delivery to the designated area and stimulate cell adherence to the wound bed. More recently, cell transplantation exploiting hydrogel carriers have gain interest among researchers. In the past decade biomaterials to mediate cell delivery and accommodate cells in a 3D microenvironment have been investigated. A plethora of synthetic and natural polymers which may form hydrogels have been studied as potential cell delivery vehicles due to their ability to integrate with healthy tissue.

### 5.4 Hydrogels

Hydrogels are defined as polymer networks with the ability to swell and absorb water within their structure. Due to their hydrophilic nature and flexibility they are very similar mechanically to human soft-tissue. Both natural and synthetic hydrogels could be considered for tissue engineering. Natural hydrogels benefit from high biological affinity and are often easily degradable, but the risk of infection transmission and difficulties with purification has increased the popularity of synthetic hydrogels.

[125, [126] Biopolymer gels can be formed out of polysaccharides or proteins. For example, polysaccharides obtained from plants (gum acacia, guar gum, starch, psyllium [127]), seaweeds (alginate, agarose, carrageenans), micro-organisms (dextran, gellan gum) or animal derived (chitosan, chitin) (hyaluronic acid) and proteins gained from animal or human tissue (collagen, fibrin, gelatin, elastin) or animal products (silk sericin, silk fibroin) [128].

#### 5.4.1 Hydrogels in burn care

Hydrogels currently available for patient care have been reviewed by many clinicians, but a skin substitute that is able to achieve complete skin regeneration has not yet been reported [79], [129], [130], [131]. However, hydrogels play a promising role in the development of next generation skin substitutes in burn care and are often used as wound dressings [132], [133], regenerative scaffolds or delivery devices for cells and therapeutic e.g. drugs, growth factors etc. Hydrogels have several characteristics to promote skin healing such as the ability to absorb and release water, which is useful in regulating burn wound exudate. Furthermore, the architecture of hydrogels can be modified to mimic the body's own extracellular matrix and their tunable mechanical properties can provide customised elasticity and flexibility [125] and make them suitable candidates for skin regeneration [66], [134], [135], [136], [137], [138], [139].

##### 5.4.1.1 Chitosan

Chitosan is a hydrophilic, non-toxic polysaccharide derived from de-acetylated chitin, obtained from crustaceans or fungi [140]. Due to its numerous advantageous characteristics such as the ability to encourage haemostasis, the ability to be modified so that it can be degraded by human enzymes and availability of a variety of formulation forms [141], chitosan hydrogels have been widely used in many biomedical applications. Topical forms of chitosan are used as wound healing stimulating dressings, for haemostasis [142], [143] and specifically for use in the treatment of burn wounds [144], [145].

Furthermore, the positive influence of chitosan on keratinocyte proliferation and adhesion has been described previously [146] and chitosan as a bio-active polymer is suggested as a promising candidate for tissue regeneration [147.

##### 5.4.1.2 Alginate

Alginate is a negatively charged polysaccharide derived from the cell walls of brown algae (seaweed) and has hydrophilic properties. Besides its widespread use in the food and paper-printing industry, it has gained much popularity as a biomaterial due to its non-immunogenicity, low cost, and simple gelation method. Alginate is FDA approved for medical applications and is commercially available as alginate based dressings such as Kaltostat® which are widely used in burn treatment [9], [148] Alginate dressings are also commonly used for the coverage of donor sites post-skin harvest and has also been successful in the treatment of paediatric burn patients [149].

##### 5.4.1.3 Fibrin

Fibrin is a protein which can be derived from human or animal blood. It can naturally form a gel and acts as a haemostatic agent in the body after tissue injury. For this reason, fibrin has been used as a sealant (fibrin glue) in the medical field [150]. For wound healing, fibrin sealants and gels have been used for the delivery of several cell types such as fibroblasts [151], [152], mesenchymal stem cells [121] and keratinocytes [153], [154], [155]. Specifically, in keratinocyte spray delivery, additional fibrin sealant seems beneficial for adhesion of the suspension to the (artificial) wound bed [40], [124], [156]. In contrast, Currie et al. performed a histological and immunohistological analysis and did not show a difference when adding fibrin glue to a keratinocyte spray delivery system in terms of epithelialisation [157].

Furthermore, fibrin has also been explored for keratinocyte transplantation in combination with a dermal substitute. For example, encapsulated keratinocytes seeded in alloderm [158], keratinocytes seeded on a fibrin based dermal matrix containing fibroblasts [153], [159] or as a glue to enhance adhesion of human dermis [160] or Integra [161]. More recently, angiogenesis stimulating factors have been added to fibrin scaffolds to improve regeneration of ischemic tissue [162].

##### 5.4.1.4 Collagen

Collagen is the most abundant protein in the human body, it is the main structural protein of the extracellular matrix and has a key role in wound healing [163]. Therefore, many tissue engineered collagen based products have been developed. In 1981, Burke and Yannas developed an artificial dermal replacement based on collagen, which has eventually led to the production of the commercialised dermal substitute Integra [164]. In the same decade, Hansbrough et al. used a collagen-glycosaminoglycan scaffold with attached cultured autologous keratinocytes and fibroblasts in burn wound treatment [97]. Since then, collagen matrices in different forms have been investigated thoroughly in wound healing; as (a)cellular dermal replacements [163], [164], [165], [166], [167], [168] or as a bilayered skin substitutes such as Orcel [166], Transcyte [169], Apligraft [170], Integra [171] and Matriderm [63]. Also, collagen hydrogels have been developed for tissue regeneration [64] with autologous cells incorporated to improve burn wound healing [139]. Although widely investigated and used in clinical practice, collagen matrices and hydrogels have a fast degradation when applied to human tissue which can have an undesirable effect. However, the rapid degradation of collagen-based biomaterials can be stabilized through chemical cross-linking [172].

Examples of other hydrogels used for cell delivery in wound healing or specifically burn care are gelatin [173], hyaluronic acid [81], [174], silk sericin [70], [175] and dextran [66], [176].

All the above mentioned hydrogels have been successfully translated to clinical practice and some are part of the standard burn wound treatment arsenal. Hydrogels have advanced burn care as part of tissue engineered skin substitutes, incorporated in dressings, topical creams or as sprayable substance.

## 6 Methods of spray deposition

### 6.1 Spray parameters and cell viability

Cell transplantation can be achieved by several techniques. In this chapter the focus lies on cell transplantation to the tissue via aerosol or spray delivery.

Sprayed cells are expected to be damaged at time of impact to the receiving surface. Following impact, the cell membrane can elongate and deform. Cell rupture and subsequently cell death can occur in largely overstretched cell membranes [177]. More precisely, cells can tolerate a cell membrane area stretch of up to 5% before it becomes detrimental to cell survival. Cell elongation, deformation and subsequent cell survival depends on many variables such as target surface characteristics, viscosity of the transporting fluid/media and velocity of the delivered cell containing droplet, nozzle distance and diameter.

Veazey et al. investigated the cell viability of xenogeneic 70% confluence fibroblasts immediately after aerosol delivery with an airbrush system and their growth behaviour in a culture model [178]. The airbrush system used could be adjusted for different nozzle diameters (312, 494, 746 μm) and air pressure at delivery (ranging from 41 kPa to 124 kPa). It was found that cell viability directly measured post aerosol delivery significantly decreased with higher pressure and smaller nozzle diameter. For cell proliferation studies, only the highest pressure with smallest nozzle diameter combination showed a delayed population doubling time and the time to reach confluence was doubled [178].

In another in vitro study with 80% confluence neonatal dermal rat fibroblasts an analytical model was proposed to describe the impact of several spray parameters in a droplet-based spray application to the cell viability. Stiffness of the tissue surface, high cell-viscosity and cell-velocity had a negative influence on cell viability post spray impact, whereas a larger cell-containing droplet diameter had a positive effect on cell viability. The latter was explained as a cushioning effect of the droplet to the surface protecting the cell within the droplet [179].

In other words, cell viability is expected to be highest in large and low-viscosity single cell-containing droplets sprayed with low-velocity onto a soft tissue surface.

Wounds would serve as a soft receiving surface for cell transplantation and can be expected to be highly viscous when hydrogels are used. Hence, tailored spray devices, with pressures and nozzle diameters optimized for cell survival can play an important role in improving cell delivery.

### 6.2 Spray systems

Spray systems are being widely used in many industries. Surprisingly little research has focused on the influence of the type of aerosol device on mammalian cell survival after transplantation.

#### 6.2.1 Low and high pressure spray nozzle

Fredriksson et al. evaluated 7 different application techniques for cell transplantation on cell viability and proliferation in an in vitro study. Based on current clinical practice they included commercially available spray systems: spray nozzle systems such as the Harvest SK/S Spray Applicator Kit®, high and low pressure Tissomat applicator in combination with a Duploject™ spray nozzle, a Duploject™ spray nozzle without additional pressure control and two non-spray systems: pipetting and paintbrushing. This study showed an approximate 50% drop in viable cell count immediately after transplantation when using a high pressure device (200 kPa) and a further decline to nearly 40% viable cell count after 2 weeks of culturing, which was comparable to the paintbrush [180]. In contrast, Harkin et al. measured a 20% higher post aerosol delivery cell survival with similar pressures [156]. The immediate cell survival is comparable with other studies utilising low pressure delivery methods/systems [178]. Although no statistically significant differences were displayed, the poorest cell viability after 2 weeks was seen in the high pressure device and paintbrush [180]. Fredriksson et al. hypothesised that an additional application of fibrin sealant might improve cell survival. Furthermore, the authors emphasized the importance of measuring the proliferation capacity of cells post aerosolisation, since a large difference was seen in their data among the different devices. Interestingly, the delivery pressure and nozzle diameter of clinically used manual cell spray devices is unclear and might impact on cell viability and proliferation capacity.

Aerosol delivery with handheld airbrush systems with adjustable air pressure supply have also been previously investigated and studies have demonstrated consistent acceptable cell viability of above 80% with low delivery pressure (below 69 kPa) [171], [178]. According to Veazey et al., this system should also be compatible with alginate-, gellan, hyaluronic and hyaluronate-based hydrogel cell carriers [68]. However, to date, there is no published data to support this statement.

#### 6.2.2 Liquid atomizer

Liquid atomizers or nebulizers originally designed for aerosol drug delivery to the trachea have also been explored for cell delivery. In burns, inhalation injury can occur with damage to the trachea and drug- or cell delivery could be used to improve the healing of these injured areas. Sosnowski et al. investigated the use of 5 different atomizers for cell delivery to the trachea in terms of cell viability. However, a droplet size below 20 μm was found to be incompatible for fibroblast encapsulation and 3 nebulizer devices had to be excluded. The nasal atomizer (NA) and Microsprayer Aerosolizer (MSA) had above 90% viable cells post spraying, but the viable cell count in the NA group declined to 65% 48 h after spraying, indicating that it was a more destructive aerosol technique [181].

All spray cell delivery techniques have been investigated in vitro or rodent studies, which has led to the development of commercial spray devices that are now available in clinical practice for burn wound treatment.

## 7 Potential therapeutic applications

### 7.1 Future approaches keratinocyte transplantation

Several reviews in the last decade have discussed the future implications of skin tissue engineering and/or specifically keratinocyte cell transplantation in the treatment of burns [35], [36], [54], [131], [182].

Larger burn wounds often require mesh grafting. Autologous epidermal cell transplantation can complement mesh grafting by stimulating rapid epithelialization, which is highly desirable to improve patient's chance of survival and eventually improve scarring. Burns specific clinical studies investigating keratinocyte transplantation are available, but due to heterogeneity of the studies and different outcome parameters the evidence remains low. Comparative trials with standardized outcomes and ideally randomized treatment for available cell transplantation techniques are required.

Due to the disadvantages of CEA sheets, future research is focused on optimizing keratinocyte proliferation by transplantation of pre- or sub confluence cells. Further improvement of keratinocyte culture method in terms of culture time, reducing infection risk and elimination of xenobiotic products and also antibiotics needs to be further investigated.

Graft attachment in keratinocyte transplantation remains an important focus for research. Boyce and Supp developed a cultured skin substitute containing cultured human keratinocytes and fibroblasts attached to a collagen-glycosaminoglycan matrix which seems to form a basement membrane at the dermal-epidermal junction in vitro [183]. Importance of basement membrane formation and rapid epithelialisation has to be taken into account in novel cell spray or carrier delivery methods [183], [184].

### 7.2 Future spray cell delivery systems for burns wound care

Spray cell delivery to burn wounds can overcome the major issues of conventional grafting techniques by reducing donor site and enhance fast re-epithelialisation. The available delivery systems can be improved by optimizing spray features to aim for high cell viability and proliferation. This should be tailored according to cell type and receiver surface. Spray features to optimise might be: air delivery pressure, nozzle designs, carrier type and depending on technique of delivery, cell containing droplet size [178], [179], [181]. Further research should take into account the importance of preventing cell damage, since this could reflect poor proliferation [178], [180]. Hydrogels could potentially serve as a mechanical protection for the cells during transplantation and provide structural support once transplanted. Although in vitro studies have shown good short term cell survival post aerosol delivery, clinical studies have not been able to show similar results as yet. The challenge for researchers is to develop a feasible spray delivery system with acceptable cell viability and proliferation which can be translated to clinical studies. Also, current clinical cell spray devices could potentially benefit from these optimized features.

## Funding

This work was supported by the Scar Free foundation, formally known as the Healing Foundation. The funding source had no involvement in designing the study, data collection, interpretation of data and decision to submit the article for publication. None of the authors has a financial interest in any of the products, devices, or drugs mentioned in this manuscript.

## Conflict of interest

The authors state no conflict of interest.

## Permission note

Permission to publish Fig. 4 and Fig. 5 have been received from Elsevier.
